# Non-operative management of a black esophagus associated with perforated gastric ulcer: A case report and literature review

**DOI:** 10.1016/j.ijscr.2025.111606

**Published:** 2025-07-03

**Authors:** Amandine Serrano, Anaïs Prelot-Claudon, Antoine Mathivet

**Affiliations:** a Department of Visceral Surgery, CHU Nîmes, Place du Pr R. Debré, 30029 Nîmes cedex 9, France; b Université de Montpellier, 163 rue Auguste Broussonnet, 34090 Montpellier, France

**Keywords:** Esophagus, Esophagitis, Perforation, Ulcer, Case report

## Abstract

**Introduction:**

Acute esophageal necrosis (AEN), or black esophagus, is a rare condition typically observed in critically ill patients. While esophageal perforation is a known complication, the coexistence of AEN with a perforated gastric ulcer has not been previously reported. Surgical management is standard in such cases due to the risk of rapid deterioration.

**Case report:**

We report the case of a 67-year-old male with multiple comorbidities, admitted for acute respiratory failure and treated in the intensive care unit. He developed hemorrhagic and septic shock secondary to AEN, with active bleeding at the cardia. Endoscopy confirmed black esophagus. A subsequent CT scan showed pneumoperitoneum and perigastric fat stranding, consistent with a perforated gastric ulcer. Despite recurrent bleeding, the patient was managed conservatively, with intravenous proton pump inhibitors, octreotide for bleeding control, broad-spectrum antibiotics, transfusions, and close clinical and radiological monitoring. No nasogastric tube was used due to esophageal necrosis. Endoscopy was repeated for hemostatic therapy and showed progressive mucosal improvement. Anticoagulation was safely resumed. The patient improved and was discharged without surgery.

**Discussion:**

Although surgical treatment is typically required in gastrointestinal perforations, especially when associated with AEN, our case demonstrates that a carefully selected patient can be managed non-operatively. The decision was based on clinical stability, imaging findings, and multidisciplinary consensus.

**Conclusion:**

This is the first reported case of AEN associated with a perforated gastric ulcer successfully treated without surgery. It suggests that non-operative management may be considered in select cases under close supervision.

## Highlights

• First reported case of black esophagus with perforated gastric ulcer.
• Entirely managed with conservative medical therapy.
• Avoidance of nasogastric tube due to esophageal necrosis.
• Endoscopic control performed despite known perforation.
• Case supports individualized surgical decision-making.

## 1 Introduction

Acute esophageal necrosis (AEN), also known as black esophagus, is a rare but severe condition characterized by diffuse black discoloration of the esophageal mucosa. It typically affects elderly, critically ill patients with multiple comorbidities, and carries a high morbidity and mortality rate. The pathogenesis is multifactorial, involving esophageal hypoperfusion, impaired mucosal defenses, and gastric reflux injury.

Although AEN has been associated with various complications, including perforation, gastrointestinal bleeding, the coexistence of AEN with a perforated gastric ulcer has not been previously described. Furthermore, such complications are usually managed surgically due to their severity and risk of deterioration. In the largest systematic review to date, published in 2020, Schizas et al. [1] reported that although most patients were managed conservatively, nearly one-quarter required surgical or endoscopic intervention, especially in severe cases or when complications occurred. This highlights the clinical dilemma in borderline situations, where the decision between surgery and conservative care must rely on individual assessment and multidisciplinary judgment.

We report here the first known case of AEN associated with a perforated gastric ulcer successfully treated with conservative medical management. This case highlights the challenges and potential of a non-operative approach in highly selected clinical situations. This study has been reported in line with the SCARE 2020 criteria [2].

## 2 Case report

We present here the case of a 67-year-old male patient, referred to the emergency department on February 26th, 2025, with dyspnea, cough and fever for one week. His medical history included hypertension, chronic obstructive pulmonary disease (COPD), atrial fibrillation treated with apixaban, and a surgically treated malignant melanoma of the left leg. Initial investigations suggested an acute exacerbation of COPD. Due to clinical severity (use of accessory respiratory muscles, tachypnea) he was transferred to the intensive care unit (ICU) for non-invasive ventilation, systemic corticosteroids and empiric antibiotic therapy with amoxicillin/clavulanate.

**Fig. 1:**
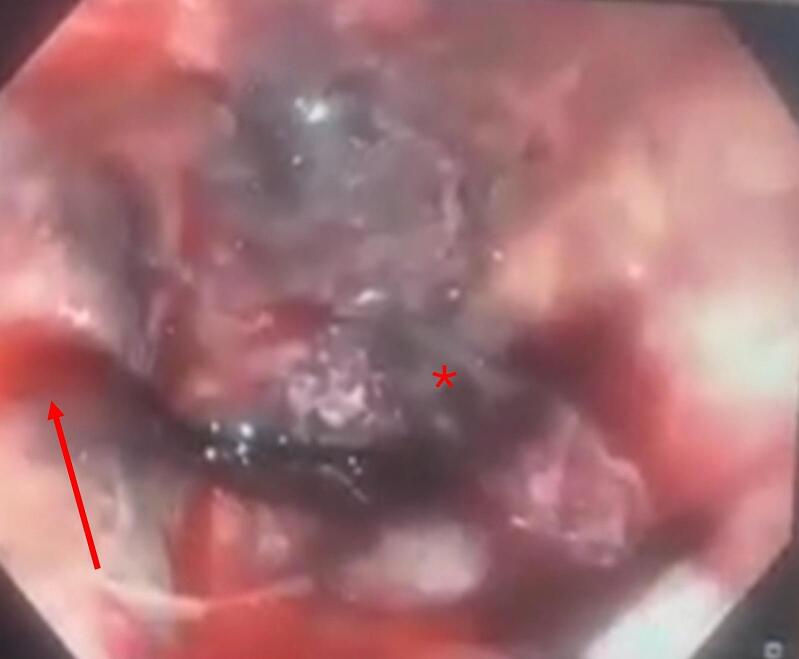
Upper endoscopy showing an aspect of black esophagus (star: necrotizing esophageal mucosa; arrow: active bleeding).

On the 1st of March, after a respiratory improvement, he developed hypotension with a 3 g/dL hemoglobin drop, rapidly progressing to hemorrhagic and septic shock requiring norepinephrine. A contrast-enhanced Computed Tomography (CT) scan revealed active bleeding in the region of the gastric cardia. Subsequent gastrointestinal endoscopy demonstrated an aspect of circumferential black discoloration of the esophageal mucosa, consistent with acute esophageal necrosis or black esophagus (Fig. 1), and active bleeding from the cardia. Hemostasis was achieved with hemostatic powder, Hemospray®, delivered through a 7 French catheter via the endoscope working channel, performed by a senior gastroenterologist with experience in upper endoscopy. Alternative diagnoses such as Mallory–Weiss tear or Boerhaave's syndrome were excluded based on the absence of vomiting, mucosal laceration, or mediastinal air. CT and endoscopy supported the diagnosis of AEN with a perforated gastric ulcer, without evidence of esophageal perforation. A conservative treatment was initiated with IV proton pump inhibitors (PPIs), broad-spectrum antibiotics (piperacillin-tazobactam, vancomycin, amikacin, caspofungin), and octreotide (600 μg per day as a continuous intravenous infusion for 5 days) to reduce splanchnic blood flow and control active bleeding.

**Fig. 2:**
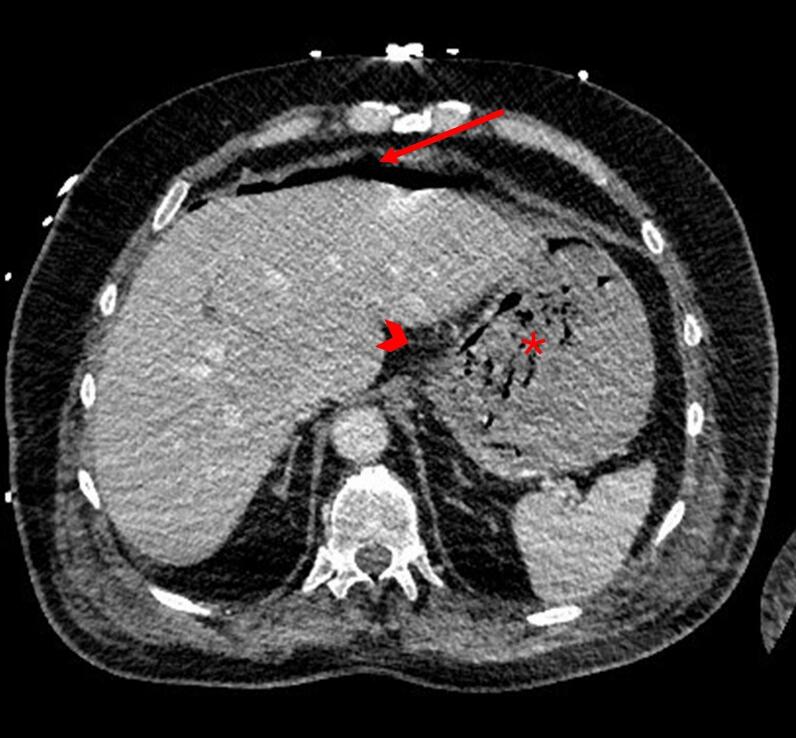
CT scan showing supra-mesocolic pneumoperitoneum (arrow), perigastric fat stranding (arrowhead) and intragastric blood (star).

**Fig. 3:**
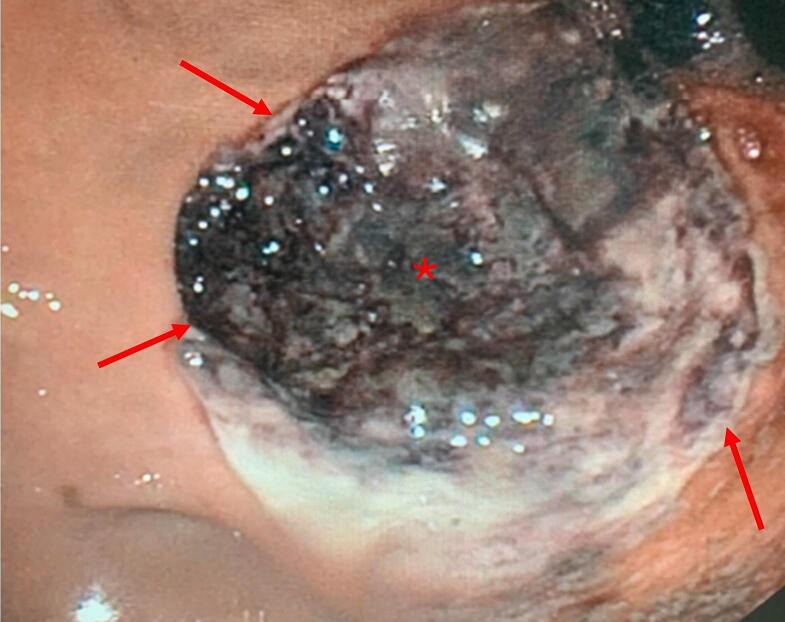
Follow-up upper endoscopy revealing a large necrotic subcardial ulcer (star), the adjacent mucosa appears normal (arrows: ulcer edge).

The patient progressively stabilized, allowing de-escalation of antibiotic therapy to piperacillin-tazobactam alone. A follow-up CT on March 3rd showed supra-mesocolic pneumoperitoneum and perigastric fat stranding, suggesting gastric ulcer perforation (Fig. 2). After multidisciplinary discussion between intensivists, surgeons, and gastroenterologists, given the limited supramesocolic free air pocket without peritoneal fluid or signs of generalized peritonitis suggesting a contained perforation, a conservative management approach was maintained, without nasogastric tube placement. This strategy was also upheld after a new episode of hematemesis and melena on the 6th of March. Repeat upper endoscopy revealed a large, necrotic, excavated subcardial ulcer with multiple exposed vessels, not amenable to endoscopic therapy (Fig. 3). Hemostatic powder was applied again to achieve temporary control of bleeding. Esophageal mucosa showed marked improvement compared to the initial examination, with partial resolution of the circumferential black discoloration.

The patient remained clinically stable under continued conservative management, supported by blood transfusions. A follow-up CT scan on March 11th showed regression of the pneumoperitoneum and a decrease in the amount of intraperitoneal free fluid. Norepinephrine gradually weaned off until March 13th. This management allowed clinical improvement and normalization of biological parameters (lactate 1,6 mmol/L vs 4,6 on March 1st, white blood cells 10,2 G/L vs 34,9 on March 1st, CRP 41 vs 84 mg/L on March 1st). The patient was then transferred to the step-down unit once stable.

**Fig. 4:**
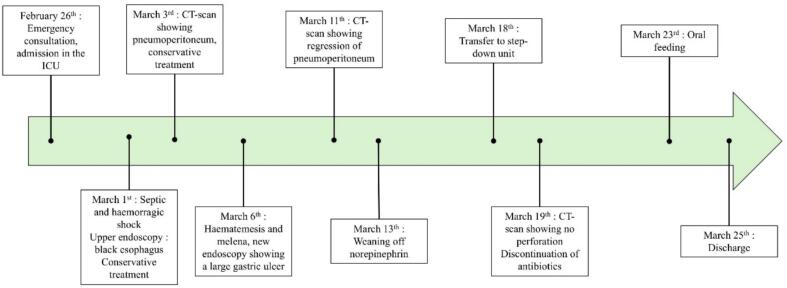
Management sequence of the patient presenting a black esophagus and a perforated gastric ulcer.

In the step-down unit, the patient's condition continued to improve gradually. CT with oral contrast on March 19th showed no extravasation. Intravenous PPIs were switched to double-dose oral long-term therapy. Piperacillin-tazobactam was discontinued after 18 days of treatment. Anticoagulation was safely resumed without bleeding recurrence. While he initially received only parenteral nutrition in the ICU, oral feeding was resumed with supplements on March 23rd, and he was discharged two days later. The entire management sequence is summarized in Fig. 4.

## 3 Discussion

We presented the case of a 67-year-old male patient who presented a hemorrhagic and septic shock due to AEN associated with a perforated gastric ulcer. Despite the initial severity and recurrence of upper gastrointestinal bleeding, the patient was successfully managed with an entirely conservative approach.

### 3.1 Acute necrotizing esophagitis: a rare but severe entity

Acute esophageal necrosis (AEN) or black esophagus [3,4] is a rare condition characterized by a circumferential black discoloration of the esophageal mucosa, typically affecting the distal third and ending at the gastroesophageal junction [5,6]. It is most often identified in critically ill patients with multiple comorbidities and is associated with significant morbidity and mortality. The incidence of AEN is estimated to be between 0.01 % and 0.2 % [[3], [4], [5], [6]] and has reached 0.28 % in a retrospective endoscopic series [7]. The diagnosis is made endoscopically by the presence of circumferential black mucosal discoloration, typically ending at the gastroesophageal junction. Histological confirmation is rarely required.

The pathophysiology of AEN is multifactorial, involving hypoperfusion, oxidative stress, impaired defenses, and gastric reflux injury [3,5,8]. The distal esophagus is a vascular “watershed” zone mainly supplied by the left gastric artery, making it vulnerable to hypoperfusion [4].

AEN mostly affects males (approximately 80 %) [4], at a mean age of 67 years [4]. Common comorbidities associated with AEN include diabetes mellitus (24 %), malignancy (20 %), hypertension (20 %), alcohol use (10 %), and coronary artery disease (9 %) [4], but we also find chronic renal or pulmonary disease, or malnutrition [[5], [6], [7]].

Although potentially reversible, AEN is often a marker of severe systemic illness, with mortality rates approaching 30–32 % [1,[3], [4], [5],^7^]. Several complications have been described, including esophageal stricture (about 25 %), mediastinitis or abscess formation, and esophageal perforation (6–7 %) [3,4]. These are associated with worsened prognosis and often necessitate interventional management.

### 3.2 Association between black esophagus and upper gastro-intestinal tract perforation

Perforation is one of the most serious and life-threatening complications of AEN, occurring in 6–7 % of cases [1,3,4,7]. It usually affects the distal esophagus early in the disease, leading to mediastinitis, abscess, empyema, or septic shock. Mortality is high, due both to the perforation and patient fragility [3,4]. Surgical intervention is generally considered mandatory in cases of esophageal perforation complicating AEN. Gurvits et al [3,4] emphasize that primary suture repair is usually not feasible because of the friability of necrotic tissue and recommend esophagectomy or wide drainage with delayed reconstruction in selected cases [4]. This approach is consistent with case reports in the literature. Wu and Ochiai both reported esophageal perforation managed surgically by esophagectomy [9,10].

Other locations of perforation have been reported less frequently. A literature review has been performed, identifying no reported case of AEN with perforated gastric ulcer. Köksal described a case of duodenal ulcer perforation in the setting of AEN, managed by laparotomy [8]. According to the authors, there is a pathogenic association between black esophagus and duodenal ulcer, because of the common blood supplies of the distal esophagus and of the first and second parts of the duodenum. Magariños described diffuse esophageal and gastric necrosis with gastric perforation, in a non-operable patient who died shortly after admission [6]. In these previously reported cases of AEN with gastrointestinal perforation, surgery was often unavoidable due to extensive contamination, mediastinitis, or hemodynamic instability. In contrast, our patient had localized signs on imaging, no diffuse peritonitis, and showed early clinical improvement. These favorable features may represent potential selection criteria for a conservative approach, though caution and close monitoring remain essential.

Although AEN has been associated with gastrointestinal perforations, none to date, to our knowledge, has described coexistence with a perforated gastric ulcer. Moreover, most previously published cases of perforation in the setting of AEN required surgical intervention, often in critical care settings. In this context, our case represents a unique clinical scenario.

### 3.3 A unique case of black esophagus with perforated gastric ulcer

To our knowledge, this is the first documented case of AEN associated with perforated gastric ulcer, successfully managed non-operatively. This rare combination posed a significant challenge for conservative management.

Initial presentation included instability, hemoglobin drop probably favored by apixaban, and imaging suggesting perforation. Such cases usually require prompt surgery, but here a non-operative strategy was selected after multidisciplinary review. This decision was based on the absence of clinical diffuse peritonitis, signs of contained perforation on imaging, and progressive clinical stabilization. It involved broad spectrum antibiotics, PPIs and a close clinical and biological management.

Managing a perforated gastric ulcer typically involves nasogastric decompression to reduce gastric pressure and limit contamination. In this case, however, the placement of a nasogastric tube was contraindicated due to mucosal fragility and risk of worsening injury from mechanical trauma or reflux [7,11]. The absence of this standard supportive measure necessitated enhanced clinical vigilance and close radiological monitoring.

Although upper endoscopy is typically contraindicated in cases of gastrointestinal perforation, in this case it was performed after multidisciplinary discussion due to ongoing bleeding. Endoscopy allowed both therapeutic intervention (hemostatic powder application) and dynamic reassessment of mucosal healing. This highlights the importance of balancing theoretical risks with the clinical need for direct visualization in selected cases.

The success of this approach was also illustrated by the safe reintroduction of curative anticoagulation, initially interrupted due to active bleeding. Anticoagulation was resumed after stabilization, without recurrence.

This case highlights the possibility of successful conservative management in highly selected patients with complex upper gastrointestinal conditions, when individualized decision-making and close multidisciplinary follow-up are ensured.

## 4 Conclusion

This case illustrates an exceptionally rare association between acute esophageal necrosis and a perforated gastric ulcer, managed successfully without surgery. It suggests that, in carefully selected patients, non-operative management may be an alternative, provided that close clinical monitoring and multidisciplinary decision-making are ensured. Additional studies are needed to better define the limits and indications of non-operative management in such complex scenarios.

## CRediT authorship contribution statement

AS: Study concept and design, data collection, writing the paper.

APC: Study concept and design, validation.

AM: Study concept and design, writing the paper, validation.

## Consent for publication

Written informed consent was obtained from the patient for publication and any accompanying images. A copy of the written consent is available for review by the Editor-in-Chief of this journal on request.

## Ethical approval

This case report does not require ethical approval as it involves a single patient case that is anonymized and does not include any identifiable personal information.

Institution : CHU Nimes

## Guarantor

Guarantor is Antoine MATHIVET (last author, corresponding author).

## Patient perspective

No direct patient statement was obtained, but the patient expressed satisfaction with the outcome when discharged home.

## Research registration number

Not applicable.

## Funding

There is no funding source for this work.

## Declaration of competing interest

The authors declare no conflict of interest related to with work.

## References

[1] Schizas D., Theochari N.A., Mylonas K.S., Kanavidis P., Spartalis E., Triantafyllou S., Economopoulos K.P., Theodorou D., Liakakos T. (2020). Acute esophageal necrosis: a systematic review and pooled analysis. World J. Gastrointest. Surg..

[2] Harvard T.H. Chan School of Public Health, Boston, USA, Kerwan A., Al-Jabir A., Mathew G., Sohrabi C., Rashid R., Franchi T., Nicola M., Agha M., Agha R., University College London Hospital, London, UK, Royal Free London NHS Foundation Trust, London, UK, Imperial College School of Medicine, London, UK, Wellington Regional Hospital, Te Whatu Ora Capital Coast and Hutt Valley, Wellington, New Zealand, Imperial College London, London, UK, Premier Science, London, UK (2025). Revised Surgical CAse REport (SCARE) guideline: An update for the age of Artificial Intelligence. PJS.

[3] Gurvits G.E., Shapsis A., Lau N., Gualtieri N., Robilotti J.G. (2007). Acute esophageal necrosis: a rare syndrome. J. Gastroenterol..

[4] Gurvits G.E. (2010). Black esophagus: acute esophageal necrosis syndrome. World J. Gastroenterol..

[5] J. Benass, S. Berrag, C. Jioua, S. Ouahid, H. Seddik, Black esophagus: a life-threatening consequence of hypoperfusion, Cureus 16 (n.d.) e75769. doi:10.7759/cureus.75769. PMC11733398 39816310

[6] Magarinos J., Akcelik A., Schmidt A., Petrov R., Bakhos C. (2023). A rare case of combined black esophagus and stomach: a case report. Ann Esophagus.

[7] Augusto F., Fernandes V., Cremers M.I., Oliveira A.P., Lobato C., Alves A.L., Pinho C., de Freitas J. (2004). Acute necrotizing esophagitis: a large retrospective case series. Endoscopy.

[8] Köksal A.Ş., Eminler A.T., Parlak E., Uslan M.I., Cücen E. (2015). Black esophagus and duodenal perforation : more than an incidental association. Acta Gastroenterol. Belg..

[9] Wu M.-H., Wu H.-Y. (2014). Incremental change in acute esophageal necrosis: report of two cases. Surg. Today.

[10] Ochiai T., Takeno S., Kawano F., Tashiro K., Nanashima A., Tsuzuki R., Doi K. (2023). Successful treatment of esophageal perforation due to black esophagus (acute esophageal necrosis): a case report. Gen Thorac Cardiovasc Surg Cases.

[11] Goldenberg S.P., Wain S.L., Marignani P. (1990). Acute necrotizing esophagitis. Gastroenterology.

